# High Seroprevalence of Antibodies against Arboviruses among Pregnant Women in Rural Caribbean Colombia in the Context of the Zika Virus Epidemic

**DOI:** 10.3390/antib9040056

**Published:** 2020-10-21

**Authors:** Elena Marbán-Castro, Germán J. Arrieta, Miguel J. Martínez, Raquel González, Azucena Bardají, Clara Menéndez, Salim Mattar

**Affiliations:** 1 ISGlobal, Hospital Clínic, Universitat de Barcelona, 08036 Barcelona, Spain; mikel.martinez@isglobal.org (M.J.M.); raquel.gonzalez@isglobal.org (R.G.); azucena.bardaji@isglobal.org (A.B.); clara.menendez@isglobal.org (C.M.); 2 Consorcio de Investigación Biomédica en Red de Epidemiología y Salud Pública (CIBERESP), 28029 Madrid, Spain; 3 Corporación Universitaria del Caribe (CECAR), Grupo de Salud Pública, Sincelejo 700001, Colombia; GERMAN.ARRIETA@cecar.edu.co; 4 Clínica Salud Social, Sincelejo 700001, Colombia; smattar@correo.unicordoba.edu.co; 5 Centro de Investigação em Saúde de Manhiça (CISM), Manhiça 1929, Mozambique; 6 Instituto de Investigaciones Biológicas del Trópico, Universidad de Córdoba, Montería 230002, Colombia

**Keywords:** zika, dengue, chikungunya, seroprevalence, Colombia

## Abstract

Mosquito-borne viruses such as dengue (DENV), chikungunya (CHIKV), and Zika (ZIKV) have spread in recent decades. We aimed to assess seroprevalence of arboviral infections in pregnant women living in Cereté, Caribbean Colombia. In 2016 a cross-sectional facility-based sero-survey study was performed among pregnant women (*N* = 90). Most of them (66%) reported at least one symptom or sign compatible with arboviral infection over the previous 15 days. All screened women had a positive IgG for DENV, 89% for ZIKV, and 82% for CHIKV. One woman tested positive for ZIKV IgM. This study shows the high exposure among pregnant women to arboviruses in endemic areas, shown by the high seroprevalence of past arboviral infections. Given the evidence on the potential risks of these arboviral infections on pregnancy and infant outcomes, these results highlight the need for continuous epidemiological surveillance of arboviral diseases, particularly among those most of risk of their harmful consequences.

## 1. Introduction

In last centuries, human epidemic mosquito-borne viruses such as Dengue (DENV), Chikungunya (CHIKV), and Zika (ZIKV) have emerged and spread worldwide, affecting a third of the human population [1]. Pathogenic arboviruses with the greatest impact on humans are ZIKV and DENV from the *Flaviviridae* family, and CHIKV from the *Togaviridae* family [1]. Although 80% of arboviral infections are asymptomatic, severe complications are experienced by pregnant women [2,3]. Consequences of DENV infection during pregnancy are an increased risk of hemorrhagic fever and shock syndrome, fetal loss, sever neonatal infections, and acute respiratory distress in infants [2]. CHIKV infection in pregnancy is associated with miscarriages, severe neonatal infection, sepsis, thrombocytopenia, rarely disseminated intravascular coagulation, cerebral edema, encephalitis, and cerebral hemorrhages [1,2]. The ZIKV epidemic of 2015–2017, resulted in thousands of poor pregnancy outcomes, such as stillbirths and miscarriages, and birth defects among neonates born to infected mothers, known as Congenital Zika Syndrome; as well as neurodevelopmental disabilities during childhood [2,4,5,6,7,8,9].

Since the beginning of the ZIKV epidemic in 2015, the National Health Institute in Colombia conducted intensified surveillance for ZIKV-associated microcephaly [10]. The first autochthonous ZIKV cases in Colombia were reported in October 2015, and the first ZIKV associated congenital case of microcephaly in April 2016 [10,11]. Only 10% of all ZIKV cases reported in Colombia, and 32.3% of pregnant women, had laboratory confirmation of infection by molecular diagnosis, a positive RT-PCR [11,12]. As well as in other endemic countries, in Colombia there might be an underreporting of ZIKV, microcephaly, and Congenital Zika Syndrome cases.

Diagnosis of arboviral infection in pregnant women does not differ from procedures in the general population [2]. Molecular diagnosis relies on reverse transcriptase polymerase chain reaction (RT-PCR) specific for each arbovirus in serum sample or cerebrospinal fluid; serological diagnosis relies on detection of IgM (for a recent infection) and seroconversion of IgG [2]. Detection of IgM in cerebrospinal fluid could be performed for all described arboviruses; and ZIKV RNA is detectable in urine samples [2].

Interpretation of positive and inconclusive serological results is challenging in an environment where other closely related viruses are prevalent, such as DENV, CHIKV, Yellow Fever, West Nile virus, Venezuelan equine encephalitis, St. Louis encephalitis virus, and Hantaviruses [10]. People living in endemic areas may have certain immunity and cross-reaction to ZIKV, DENV, and CHIKV immunological tests, causing false-positive results [13,14]; and confirmation of arboviral infection by microneutralization or plaque reduction neutralization test is simply unavailable in many endemic settings, where they are most needed. According to de Ory et al., the best definition of a clinical case of ZIKV in endemic areas is the clinical diagnosis based on rash with pruritus or conjunctival hyperemia and absence of other manifestations [15]. There are difficulties in the laboratory diagnosis of individual cases, especially due to cross-reactions between flaviviruses; the main problem being the evaluation of serological ZIKV assays for correct classification of cases [15].

Recommendations for pregnancies at risk include a complete screening exam for various arboviral infections to guide case determination; especially for a prompt diagnosis of ZIKV. Epidemiological and clinical studies are needed to evaluate the burden of these infections in vulnerable populations, such as pregnant women and children, to estimate the seroprevalence of several infections in the community and to guide the development of future serological diagnostic tools. In this study, we aimed to assess the seroprevalence of ZIKV, DENV, and CHIKV infections among pregnant women, in an area of South America where these arboviral infections co-exist.

## 2. Materials and Methods

This was a cross-sectional study conducted in Cereté, Córdoba state, a tropical area in North-East Colombia. All pregnant women, regardless the presence of signs or symptoms compatible with arboviral disease, were invited to attend a local health center to participate in the study. The call was performed by a local radio program, and by health professionals in peripheral health centers. All pregnant women that attended the health center in May of 2016 were invited to participate in the study. Inclusion criteria was defined as being pregnant and living in the study area. Written informed consent was obtained previous to enrollment.

Clinical, obstetrical and epidemiological data were collected into a standardized questionnaire. Serum samples were collected and shipped to the Microbiology laboratory of Hospital Clínic Barcelona for serological analysis of ZIKV, DENV, and CHIKV. Antibodies against ZIKV (IgG and IgM, using the non-structural NS 1 protein as an antigen) were detected by a commercial ELISA test (Euroimmun, Lübeck, Germany), according to the manufacturer’s instructions (negative, if the index value was <0.8; inconclusive, ≥0.8–<1.1; positive, ≥1.1). This test has shown lower cross reactivity with other antibodies against flaviviruses [15,16], and specificities ranging from 70% to 97% in different studies [17,18]. Anti-DENV IgG antibodies were tested by a widely used commercial assay (Panbio ELISA, Alere, Australia), with a sensitivity of 91.4%–96.3% and a specificity of 80.9%–87.1% [19]. Anti-CHIKV IgG were tested using a commercial immunofluorescence assay (IIFT) (Euroimmun, Lübeck, Germany) with a reported sensitivity and specificity >95% [20].

Data were entered in an Excel database. Data cleaning and analysis was performed using Stata (StataCorp LLC 2017, College Station, TX, USA). Categorical data were summarized by frequencies and percentages. Adolescence was defined by WHO standards (<19 years of age). According to the National Regulatory system in Colombia, the social stratus is a classification that groups households in six different levels according to housing and environmental characteristics. The most vulnerable groups are those included in the first three social class levels (one, two, and three).

All study procedures were performed in accordance with the ethical standards of the responsible committee on human experimentation in Colombia (Comité de ética en Investigación—Universidad de Córdoba, Acta No.10-2016), and in Spain (Comité de Ética de la Investigación con medicamentos del Hospital Clínic de Barcelona, Nº HCB/2019/0966), and with the Helsinki Declaration of 1975, as revised in 1983.

## 3. Results

All pregnant women invited to participate in the study (*N* = 90) accepted to be enrolled. The question about previous miscarriages was not responded by two participants (two missing values). Thirty-one women (34%) did not report compatible symptoms with arboviral disease in past 15 days. History of symptoms is detailed from the 59 women who recalled having suffered at least one compatible symptom. Table 1 shows characteristics of study women.

The average age of participants was 22.2 ± 5.0 years old (SD 5.0). Twenty six percent of women enrolled were adolescents. The majority of participants were recruited during second (52%) or third trimester of pregnancy (34%). All of them belonged to the lowest socio-economic status, according to National official strata division (strata 1, 2, and 3). None of them presented clinical signs or symptoms at recruitment. History of compatible symptoms in past 15 days was reported in 59 of 61 (97%) women who answered the question; 67% of all women declared to have experienced an arboviral disease when they were directly asked about Dengue, Zika, or Chikungunya disease.

One pregnant woman had a recent asymptomatic Zika virus infection, diagnosed by a positive result for ZIKV IgM, (ELISA Index Value of 1.68) and IgG (ELISA Index Value of 400), with no reported compatible clinical symptoms, and negative results for CHIKV IgG. Eighty samples (89%) were positive for ZIKV IgG, 90 samples (100%) for DENV IgG, and 74 samples (82%) were positive for CHIKV IgG. There were inconclusive results in both tests for ZIKV, IgM (one case), and IgG tests (five cases). Table 2 illustrates laboratory screening results for pregnant women.

All participants showed evidence of past DENV infection. Sixty-seven women had positive IgG results for all three arboviruses tested; there were 15 women with either CHIKV or ZIKV past infection, three women with negative results for both diseases, and five women with CHIKV past infection and inconclusive results for ZIKV. Laboratory results interpretation and its clinical significance is detailed in Table 3.

## 4. Discussion

In our study, we found a very high prevalence of past arboviral infections in pregnant women living in a town in rural Caribbean Colombia. It is consistent with reports of first ZIKV cases detected in Colombia in late 2015 [10], and stresses how the epidemics of CHIKV (2014) and ZIKV (2015) joined DENV endemic circulation and hit that specific Caribbean area. The majority of participants showed specific antibodies that evidenced previous infection. Additionally, one pregnant woman was diagnosed with a recent Zika virus infection. Interestingly, high CHIKV IgG seroprevalence suggests a massive circulation of the virus in study area previous to 2016. The first reports of CHIKV in Colombia date of 2014 [21].

Most of the participants in our study (97%) recalled having recently experienced at least one symptom compatible with arboviral disease; though only 67% of them declared to have experienced any arboviral disease when directly questioned about Dengue, Zika, or Chikungunya. Clinical signs and symptoms from these three diseases can be easily confused, posing a significant challenge for clinical diagnosis [22]. Differentiation of these infections represents a more complicated task among pregnant women for the increased risks of developing severe consequences; thus, increasing the importance of developing serological tools for case ascertainment [22].

A recent systematic review showed that only 15.7% of serological studies are conducted among pregnant women [23]. The seroprevalence of DENV in pregnant women ranged from 1% to 100% in 17 studies found, being 100% in a study performed in the Caribbean Islands of St Kitts Nevis and Jamaica [23]. This systemic review concluded that the highest seroprevalence rates of DENV IgG performed by ELISA were observed in the Caribbean region specifically, and in the Americas [20]. The prevalence of CHIKV IgG by ELISA was high in a study conducted in Thailand with pregnant women showing a seroprevalence of 71.2% [23]. From the seroprevalence studies reported in the meta-analysis, no results were specifically shown for ZIKV seroprevalence in pregnant women [20].

This concomitant circulation of arboviruses represents a major public health and biomedical challenge in many endemic settings [23]. Serological studies provide the most direct measurement to estimate the immunological landscape of infectious diseases within a population; but difficulties in the implementation of its methodology still represent gaps in knowledge of the geographic distribution of ZIKV, DENV, and CHIKV [24]. The serological tools used for detecting antibodies against DENV and ZIKV (ELISA), and anti-CHIKV (IIFT) have previously shown acceptable performance for clinical diagnosis. Although cross-reactivity between antibodies against different flaviviruses cannot be excluded, we used an ELISA test detecting antibodies against ZIKV NS1 protein that has been shown to be more specific than the widely used immunofluorescence assays [16,17].

DENV and ZIKV show a close evolutionary relationship, reflected by the high sequence conservation of both structural and non-structural proteins [24]. In this aspect, a recent published study has highlighted the importance of the identification of monoclonal antibodies (mAbs) able to react specifically with DENV or cross-react with ZIKV proteins a relevant feature for the validation of the diagnostic tools based on the NS1 protein [24]. Diagnostic tools able to differentiate DENV, ZIKV, and CHIKV infections are needed [24]; equally important is that these serological tools are developed and commercialized affordably for endemic areas, basically tropical areas in Central and South American countries.

Findings highlighted the importance of including the study of prevalent diseases in the community in health education lectures from an early age. Health prevention and education campaigns are needed to be implemented in order to increase community awareness, and political determination to implement vector-control activities to reduce arboviral infections in affected areas. However, responsibility to perform vector-control activities should not fall to citizens’ will, knowledge, capacities, or access to preventive measures. In this study, we found a very high rate (26%) of adolescent pregnancies in the community, which represents an increasing public health problem in Colombia, and other Central and South American countries, that needs further consideration from a global health perspective [25]. These pregnant adolescents are especially vulnerable to access appropriate health care services, including fewer antenatal care visits compared to adult pregnant women and resulting in poorer maternal and child health [25]. The importance of a prompt diagnosis of arboviral infection during pregnancy lies on the psychosocial impact it has for affected women’s mental health and emotional state [26,27].

Our study has several limitations. First, sample size was small; a selection bias of participants could occur, as only those who were aware of this call, by already attending antenatal care services or having a radio, could find out information of the study to attend the health center. Secondly, plaque reduction neutralization tests were not available to confirm serological results, and molecular diagnosis was not performed; thus, minimizing the chances of detecting active arboviral infection. Serological diagnosis of IgM antibodies for DENV and CHIKV was not performed due to limited availability of resources, and prioritization of testing anti-ZIKV IgM. These limitations are inherent to the study context and represents a challenge for many low- and middle-income countries, where infections are most prevalent. Finally, recall bias of symptoms could have affected history of symptoms reporting. Cross-reaction with other antigenically related flaviviruses could be happening and hampering serological diagnosis approaches.

As there is still no vaccine to prevent dramatic consequences of arboviral infections in pregnancy [28,29,30,31], rapid, cheap, and reliable serological diagnostic tools for ZIKV are of urgent importance due to the high rates of asymptomatic infections, and lack of adequate sensitivity, and selectivity, of immunoassays to detect active infections in endemic areas. ZIKV vaccine candidates will be a critical tool for prevention of ZIKV infection during pregnancy, thus their participation in vaccine trials must be enhanced [31].

Based on the ongoing risk of arboviral infections in the American continent, this study contributes to raising public awareness of mosquito-borne viruses, to support future control strategies, and recommends a continuous and close surveillance for arboviral infections. Larger-scale nationwide studies are recommended to gain a broader view of the potential threat of arboviruses among vulnerable populations, such as pregnant women and their children.

## 5. Conclusions

Exposure to arboviral infections was very high among pregnant women living in rural Caribbean Colombia. Pregnant women had a high seroprevalence of past arboviral infections to Zika, Dengue, and Chikungunya. Given that these infections often occur sub-clinically, and considering the evidence on the potential risks of arboviral infections on pregnancy and infant outcomes, these results highlight the need for continuous epidemiological surveillance of arboviral diseases, particularly among most at risk populations.

## Figures and Tables

**Table 1:** Characteristics of 90 pregnant women enrolled in the study in Cereté, Colombia, 2016.

Characteristics	Average ± SD (*N* = 90)	n/*N*	(%)
Age (years)	22.2 ± 5.0 (90)		
Gestational age (weeks)	23.3 ± 8.3 (90)		
**Age (years)**			
<19		23/90	26
19–24		40/90	44
≥25		27/90	30
**Gestational age**			
First trimester (0–12 weeks)		12/90	13
Second trimester (13–36 weeks)		47/90	52
Third trimester (27 weeks until delivery)		31/90	34
**Parity**			
Primigravidae		26/90	29
Secundigravidae		33/90	37
Multigravidae		31/90	34
**History of miscarriage**			
Yes		9/88	10
**History of signs/symptoms compatible with arboviruses in the past 15 days**			
None		2/61	3
Rash		48/59	81
Fever		52/59	88
Headache		39/59	66
Retro-ocular pain		20/59	34
Conjunctivitis		10/59	17
Myalgia		21/59	36
Arthralgia		33/59	56
**History of past arboviral disease**		60/90	67

±SD: standard deviation; n: number of participants for disaggregated variable; N: total number of participants included in each variable.

**Table 2:** Laboratory results of serum from pregnant women enrolled in the study in Cereté, Colombia, 2016.

Laboratory Screening	Laboratory Results	*n*/*N* (%)
ZIKV IgG	Positive	80/90 (89%)
	Inconclusive	5/90 (6%)
ZIKV IgM	Positive	1/90 (1%)
	Inconclusive	1/90 (1%)
DENV IgG	Positive	90/90 (100%)
CHIKV IgG	Positive	74/90 (82%)

ZIKV: Zika virus, Ig: immunoglobulin, ELISA: enzyme-linked immunosorbent assay, DENV: Dengue virus, CHIKV: Chikungunya virus, n: number of positive samples, N: number of total number of samples tested.

**Table 3:** Laboratory results interpretation.

Manufacturer	Product Name	IgG Class	Laboratory Results	Interpretation
Euroimmun	EUROIMMUM ELISA Kit Anti-Zika virus	IgG	Positive	Probable past ZIKV infection
			Negative	No evidence of past ZIKV infection
Euroimmun	EUROIMMUM ELISA Kit Anti-Zika virus	IgM	Positive	Probable recent ZIKV infection
			Negative	No evidence of recent ZIKV infection
Alere	Panbio dengue viruscapture ELISA	IgG	Positive	Probable past DENV infection
			Negative	No evidence of past DENV infection
Euroimmun	EUROIMMUM Kit Anti-Chikungunya virus IIFT	IgG	Positive	Past CHIKV infection
			Negative	No evidence of past CHIKV infection

ZIKV: Zika virus, Ig: immunoglobulin, ELISA: enzyme-linked immunosorbent assay, DENV: Dengue virus, CHIKV: Chikungunya virus; IIFT: indirect immunofluorescence test.
